# Self-assembled Messenger RNA Nanoparticles (mRNA-NPs) for Efficient Gene Expression

**DOI:** 10.1038/srep12737

**Published:** 2015-08-03

**Authors:** Hyejin Kim, Yongkuk Park, Jong Bum Lee

**Affiliations:** 1 Department of Chemical Engineering, University of Seoul, Seoul 130-743, Korea

## Abstract

Although mRNA has several advantages over plasmid DNA when delivered into cells for gene expression, mRNA transfection is a very rare occurrence in gene delivery. This is mainly because of the labile nature of RNA, resulting in a low expression level of the desired protein. In this study, self-assembled mRNA nanoparticles (mRNA-NPs) packed with multiple repeats of mRNA were synthesized to achieve efficient gene expression. This approach required only a one-step process to synthesize particles with a minimal amount of plasmid DNA to produce the RNA transcripts via rolling circle transcription. Moreover, there are no concerns for cytotoxicity which can be caused by chemical condensates because mRNA-NPs are made entirely of mRNA. An examination of the cells transfected with the mRNA-NPs encoding the green fluorescence protein (GFP) confirmed that the mRNA-NPs can be used as a novel platform for effective gene delivery.

Nucleic acids have attracted considerable attention recently as both a research tool and a potential therapeutic material for the treatment of a wide range of diseases, such as infectious diseases, cancer and inflammation. Nucleic acid-based therapeutics can be classified into two major categories: those that inhibit the production of deleterious proteins and those that enhance gene expression^1^,^2^. A range of nucleic acid-based drugs, such as microRNAs, small interfering RNAs, small hairpin RNAs and antisense oligonucleotides, are used for messenger RNA (mRNA) degradation and the inhibition of protein translation^3^, whereas the delivery of plasmid DNA is considered the most effective way of enhancing protein expression^4^,^5^. Therefore, plasmids are widely used for the development of vaccines^6^,^7^,^8^,^9^. On the other hand, plasmid DNA still requires an access to the nucleus to properly perform its function, which is a major reason for the reduced gene expression efficiency of the delivered plasmid DNA^10^,^11^. To address this issue, a range of strategies have been developed to introduce foreign genes into the host cells^12^,^13^,^14^,^15^. A number of studies have reported efficient gene expression by delivering plasmid DNA encoding the desired proteins, but they still suffer from low expression efficiency^16^.

The delivery of mRNA is another direction for the production of therapeutic proteins with several advantages over plasmid delivery^17^. First, mRNA transfection is a promising approach to gene expression without a risk of integration into the host genome. Second, there is no need for nuclear localization and transcription. These advantages can result in rapid expression and cell cycle-independent transfection, which is essential for non-dividing cells^18^,^19^. On the other hand, there have been few studies concerning the transfection of mRNA because mRNA has two major limitations: instability under physiological conditions and immunogenicity^20^. Therefore, an effective mRNA delivery system is needed to solve the aforementioned issues. To date, several non-viral vectors have been applied to the safe delivery of mRNA, such as polyplexed nanomicelles^21^, lipoplexes or liposomes^22^,^23^,^24^, dendrimers^25^, and gold nanoparticles^26^. In addition, several mRNA modification methods have been reported to be effective strategies for reducing their immunogenicity^17^,^27^,^28^. Although these approaches have been promising, a more efficient method for the delivery of mRNA into living systems is needed before the potential of mRNA-based therapeutics can be realized^29^.

By taking advantage of rolling circle replication methods^30^,^31^,^32^, previous studies showed that extremely long DNA or RNA strands can self-assemble into microstructures^33^,^34^,^35^,^36^. Inspired by these demonstrations, this paper reports a novel strategy for efficient mRNA delivery by generating nanoparticles comprised of mRNA strands with high potency for the production of proteins in cells. To obtain nano-sized particles made of RNA strands, rolling circle transcription (RCT) was performed on the plasmid DNA, driving the self-assembly of mRNA (Fig. 1). The enhanced stability of mRNA and successful expression of green fluorescence protein (GFP) as a model protein were achieved by packing strands of repeating mRNAs into nanoscopic particles. This approach also required only a one-step process to synthesize particles with minimal plasmid DNA to produce RNA transcripts via RCT for efficient delivery and high cargo capacity. An enzymatic approach, rolling circle transcription, was used to polymerize the RNA to produce condensed mRNA nanostructures that contain repeated mRNA sequences for the expression of GFP.

## Results

### Synthesis of mRNA nanoparticles

Using rolling circle replication, a plasmid was used as a circular DNA template to transcribe the mRNA strands. For this, GFP–encoding plasmid DNA (pIDTSMART-GFP) containing the promoter region for T7 polymerase was designed, as shown in the map of the plasmid DNA (see Supplementary Fig. S1 online). The template DNA also contains the eukaryotic ribosomal binding sequence (RBS), known as the Kozak sequence, which is essential for initiating translation by binding the eukaryotic ribosome to the mRNA strands and the GFP-encoding sequence between the start and stop codons. By RCT, extremely long RNA strands including repeated protein-coding sequences can be self-assembled to form nanoscopic particles which are termed messenger nanoparticles (mRNA-NPs) made from messenger RNA.

**Figure 2:**
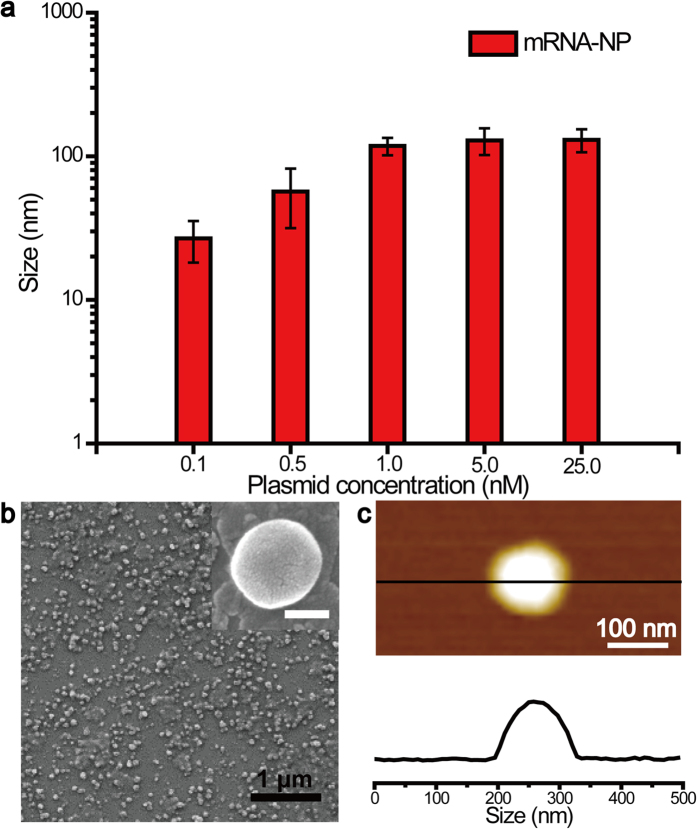
(**a**) Size distributions of the mRNA-NPs transcribed from different concentrations of GFP-encoding plasmid DNA template. (**b**) Scanning electron microscopy image (inset: scale bar, 100 nm) and (**c**) atomic force microscopy image of the mRNA-NP-5 and cross-sectional measurement along the line shown in panel.

The optimal concentration of plasmid DNA was determined by dynamic light scattering (DLS) analysis of each sample generated from the RCT reaction with different amounts of plasmid DNA. As shown in Fig. 2a, the size of the resulting mRNA-NPs (30 to 200 nm) tended to increase with increasing concentration of plasmid DNA template in the RCT reaction mixture because of the higher rates of RNA generation. In addition, there was a threshold level of template DNA needed to produce nanoparticles that was between 0.05 nM and 0.1 nM, since nanoparticles did not form with 0.05 nM plasmid DNA. The mRNA-NPs transcribed with plasmid DNA concentrations of 1 and 5 nM, approximately 120 nm in size, were examined further. (Herein, mRNA-NPs generated from 1 and 5 nM of plasmid DNA are referred to as mRNA-NP-1 and mRNA-NP-5, respectively.) The optimal concentrations of rNTPs, T7 polymerase and plasmid DNA for the RCT reaction were also examined by gel electrophoresis (see Supplementary Fig. S2 online).

### Characterization of mRNA-NPs

The resulting nanoparticles were observed by scanning electron microscopy (SEM). The mRNA-NPs had a spherical shape with diameters ranging from 100 to 200 nm (Fig. 2b and see Supplementary Fig. S3 online). Atomic force microscopy (AFM) also revealed the spherical structure of the particles (Fig. 2c), and the size of the mRNA-NPs in the AFM image was consistent with the SEM and DLS results.

**Figure 3:**
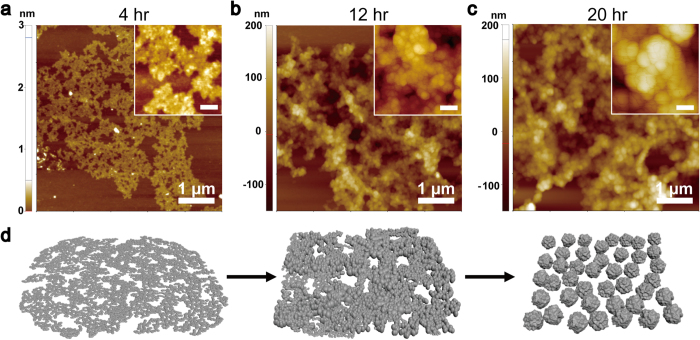
Time-course of the fabrication of mRNA-NPs observed with AFM. The RCT reaction was carried out at 37 °C for 4 h (**a**), 12 h (**b**) and 20 h (**c**). Inset: 100 nm. (**d**) Schematic diagram of mRNA-NP synthesis. Multiple copies of mRNA strands were first entangled. As the mRNA strands grow longer, they begin to form small spherical structures. The spherical structures gradually grow larger, and finally become 120 nm-sized mRNA-NPs.

To examine the formation of mRNA-NPs over time, the RCT products were observed by AFM after a RCT reaction time of 4 h, 12 h and 20 h (Fig. 3). As shown in Fig. 3a, long repeats of the mRNA strands were first entangled with each other in a similar manner to the entanglement of long random-sequence single-stranded DNA^36^,^37^,^38^. A higher magnification image and cross-sectional analysis showed that the height of the entangled mRNA strands was only approximately 2 nm (see Supplementary Fig. S4 online), indicating that RCT products have a two dimensional structure at this stage of the reaction. After further reaction time, the 2-D structure grew thicker and began to form some small spherical structures (Fig. 3b) because of the increased inhomogeneity of the spatial distribution of mRNA strands caused by the greater entanglement and twisting^39^. At this stage of the reaction, the RCT products began to adopt three-dimensional structures with the formation of interconnected bead-like small particles with diameters of approximately 30 nm. The bead-like small particles grew larger with increasing reaction time, finally resulting in 200-nm globular structures (Fig. 3c). The high magnification image after 20 h of the RCT reaction showed them to be separated into individual mRNA-NPs. Figure 3d suggests a schematic illustration of the synthesis of mRNA-NPs based on time-dependent experiments.

**Figure 4:**
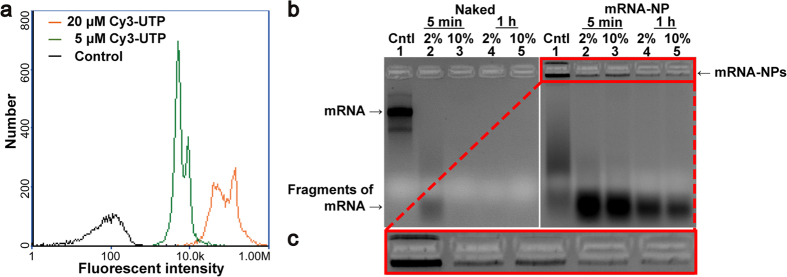
(**a**) Histograms showing the orange fluorescence intensity of the Cy3-UTP labeled mRNA-NPs. The histograms indicate mRNA-NP-1 with no Cy3-UTP (black; control), 5 μM (green) and 20 μM (orange) of Cy3-UTP, respectively. (**b**) Gel electrophoresis result of the capped mRNA encoding Xef-1 (1800 bp; left) and mRNA-NPs (right) incubated with 2% FBS for 5 min (lane 2), 10% FBS for 5 min (lane 3), 2% FBS for 1 h (lane 4), 10% FBS for 1 h (lane 5) at 37 °C or left untreated (lane 1). The naked mRNAs were destroyed within 5 min when exposed to 10% FBS. The mRNA-NPs were detected within wells and the intensities of these bands decreased when treated with serum nucleases. (**c**) High magnification image of the highlighted area in b.

The resulting particles were analyzed by gel electrophoresis to confirm that they were composed of RNA strands (see Supplementary Fig. S5 online). The resulting self-assembled mRNA-NPs could be labeled with a number of cyanine 3-labeled UTPs (Cy3-UTPs) emitting orange fluorescence via RCT reaction with the involvement of Cy3-UTPs. Therefore, Cy3-labeled mRNA-NPs were readily visible under ultraviolet light without further staining after gel electrophoresis (see Supplementary Fig. S5 online), suggesting that the mRNA-NPs were composed of RNA strands. Image cytometry was also performed to confirm that the resulting mRNA-NPs were comprised of mRNA from RCT by monitoring the change in the fluorescence intensities of the mRNA-NPs with different amounts of Cy3-UTP involved in the RCT reaction. As expected, the resulting mRNA-NPs exhibited strong fluorescence intensity after the RCT reaction with Cy3-UTP. The intensity increased significantly with increasing Cy3-UTP concentration in the reaction solution, which is consistent with the gel electrophoresis data (Fig. 4a and Fig. S5 in the Supporting Information). As a control, the mRNA-NPs were transcribed without Cy3-UTP and did not show significant fluorescent intensity (Fig. 4a).

### Stability of mRNA-NPs

The degradation of mRNA-NP and capped-mRNA were analyzed after a treatment with the nuclease-containing serum^40^ for 5 min and 1 h. Gel electrophoresis revealed significant degradation of capped-mRNA to small fragments after 5 min exposure to 2% fetal bovine serum (FBS) (Fig. 4b). In addition, a complete degradation of the free mRNA from nuclease activity was observed after 5 min incubation with 2% FBS. On the other hand, mRNA existing in nanoparticle form showed significantly enhanced resistance to nuclease after the same treatments with the serum containing media. Even after 1 h exposure to 10% serum, the majority of the mRNA-NPs were still trapped in the loading wells^41^, suggesting that the products were still in the particle formation stage. DLS confirmed that the self-assembled mRNA as a particle was relatively stable in serum containing media (see Supplementary Table S1 online).

### Cellular protein expression

For cellular protein expression, the mRNA-NPs were first treated with the transfection reagent, TransIT-X2 Dynamic Delivery System (TransIT-X2), to effectively deliver them into the cells. Different amounts of mRNA-NPs were tested to determine the optimal conditions for the mRNA-NPs to be coated with the reagent. The zeta potentials of mRNA-NPs, TransIT-X2 and mRNA-NP after treatment with TransIT-X2 were analyzed to confirm the efficient surface modification of mRNA-NPs (see Supplementary Fig. S6 online). As expected, the mRNA-NPs were coated easily with a positively-charged transfection reagent because of their high charge density. A significant change in the zeta potential from −19.1 mV (mRNA-NP) to +11.4 mV (TransIT-X2 coated mRNA-NP) was observed, indicating the successful assembly of mRNA-NPs with the reagent (see Supplementary Fig. S6 online). The size distribution was also examined by DLS, which showed a 5 nm increase in diameter after treatment with TransIT-X2 (see Supplementary Fig. S7 online).

**Figure 5:**
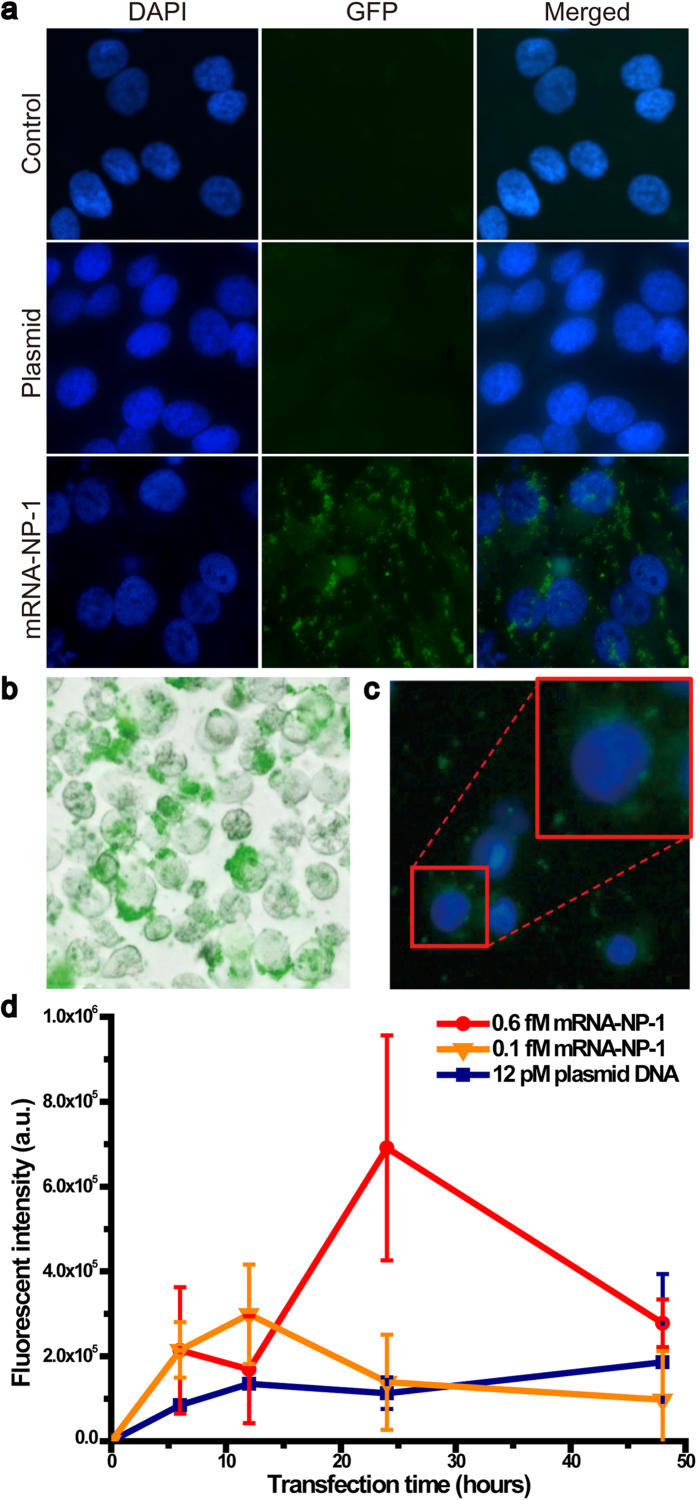
(**a**) Fluorescent microscopy images of the transfected PC-3 cells. DAPI (blue) identifies the nuclear location and the green signal provides a confirmation of transfection. The cells were transfected with 12 pM of plasmid DNA (middle) and 0.6 fM of mRNA-NP-1 (bottom) for 24 h. The control cells (top) were grown in the same condition. Merged bright field and GFP image (**b**) and merged DAPI and GFP image (**c**) of the cells transfected with 0.1 fM of the mRNA-NP-5 for 24 hours (**b**,**c**). (**d**) Mean GFP fluorescence intensities measured as a function of transfection time. The PC-3 cells were transfected with 0.6 fM (red) and 0.1 fM (orange) of mRNA-NP-1, respectively. For the control group, 12 pM (blue) of plasmid DNA was transfected into PC-3 cells. For transfection of plasmid DNA and mRNA-NPs, transfection agent was used.

Based on molecular weight analysis (see Supplementary Table S2 online), different concentrations of particles were incubated with the PC-3 cells to confirm a subcellular transfection of the coated mRNA-NP-1 and expression of GFP. The mRNA-NP-1-transfected PC-3 cells exhibited strong green fluorescence in the fluorescent microscopic images at 24 hours post-transfection (Fig. 5a, bottom) without affecting the cell viability (see Supplementary Fig. S8 online), whereas the control cells (Fig. 5a, top) showed no green fluorescence. As a control, only the pIDTSMART-GFP plasmid was also transfected. As expected, no significant green fluorescence was observed in the cells transfected with low concentrations of pIDTSMART-GFP plasmid DNA (the same amount for generating the mRNA-NP-1), confirming that GFP had been expressed from mRNA-NPs, and not from the plasmid DNA potentially entrapped within the mRNA-NPs in the mRNA-NP-transfected cells (Fig. 5a, middle). The cells treated with mRNA-NP-1 from a scrambled sequence also showed no green fluorescence (see Supplementary Fig. S9 online). Cytometry analysis revealed the maximum GFP mean intensity at 24 h after transfecting 0.6 fM of the mRNA-NP-1 (Fig. 5d). This is an approximately 6-fold enhancement in fluorescence compared to the cells transfected with only the plasmid DNA. The mRNA-NPs prepared with a fivefold higher concentration of plasmid DNA, mRNA-NP-5, could also produce significantly higher green fluorescence in the cell, indicating efficient GFP expression (Fig. 5b,c).

## Discussion

mRNA nanoparticles were synthesized via RCT. By rolling circle transcription, extremely long RNA strands including repeated protein-coding sequences can be self-assembled to form nanoscopic particles. Size distribution analysis indicated that the size of the mRNA-NPs increased with increasing concentration of plasmid DNA added to RCT (Fig. 2a). On the other hand, plasmid concentrations less than 0.05 nM resulted in no detectable particles, suggesting that the concentration of RNA strands synthesized via transcription is critical to generate the particle form. As one of the purposes was to deliver as many copies of mRNAs as possible while minimizing the use of plasmid DNA, the mRNA-NPs, approximately 120 nm in size, transcribed with 1 and 5 nM of plasmid DNA were examined further.

SEM and AFM (Fig. 2b,c) clearly show that the multi-mer mRNA had been successfully assembled into the mRNA-NPs. Furthermore, time-course experiments (Fig. 3) provide an evidence that the mRNA strands were generated continuously during RCT. Labelling with Cy3-UTP confirmed that the resulting nano-sized particle formulation after 20 h of RCT consisted of RNA strands (Fig. 4a). Before introducing mRNA-NPs to cells, this study examined whether mRNA-NPs could be protected from nuclease digestion, which is one of the major obstacles to mRNA-based gene delivery to determine the susceptibility of the mRNA particles to nuclease. After a treatment with FBS, a majority of the mRNA-NPs remained intact while the naked mRNA strands were undetectable (Fig. 4b,c), suggesting that the mRNA-NPs were protected from immediate nuclease digestion.

Due to their increased nuclease resistance, the mRNA-NPs allow for prolonged GFP mRNA translation leading to higher GFP expression. In traditional delivery methods such as lipoplexes or polyplexes complexed with naked mRNA strands, protein expression was generally sustained for as little as 6 to 10 hours due to the labile nature of the mRNA^42^,^43^. In comparison, the mRNA-NPs slowly undergo translation in the cell, and show continuous gene expression over a longer period of time. (Fig. 5). This shows that the prolonged synthesis of the desired protein is feasible by delivering mRNA-NPs and confirms that the mRNA-NPs can be used as a powerful platform for gene expression.

Protein expression involving mRNA is only transient. This feature can be advantageous for vaccination with mRNA as an example because of the noticeably lower immune response caused by mRNA than DNA or proteins^44^. Compared to the delivery of plasmid DNA, the possibility of repeated dosing while avoiding the risks caused by integration into the host genome is also a significant advantage of mRNA delivery. Furthermore, the production of two desired proteins simultaneously is possible in the case of using plasmid DNA encoding two different types of proteins as a template for the synthesis of mRNA-NPs. With all these benefits of utilizing mRNA-NPs, it is expected that the mRNA-NP approach will allow the labile nature of mRNA to be overcome in future clinical developments.

## Methods

### Plasmid DNA construction

pIDTSMART-AMP (Integrated DNA Technologies) encoding a green fluorescent protein (GFP) gene under the control of T7 promoter was used for plasmid DNA transfection and the *in vitro*-transcription of mRNA-NPs.

### Synthesis of mRNA-NPs

For the synthesis of mRNA-NPs, pIDTSMART-AMP (final concentration of 1 nM for mRNA-NP-1 and 5 nM for mRNA-NP-5) was mixed with 4 mM of ribonucleotide solution mix (Bioline), RNAPol Reaction Buffer (final concentration of 2X) and 50 units ml^−1^ of T7 RNA polymerase (New England Biolabs). For the RCT process, the reaction solution was incubated for 20 h at 37 °C. The *in vitro* transcribed mRNA-NPs were characterized by gel electrophoresis. The molecular weight of the mRNA-NP was determined by the fluorescence-based determination of mass concentration and image cytometry analysis (see Supplementary Table S2 online).

### Characterization of mRNA-NPs

An XL30-FEG (FEI) environmental scanning electron microscope and Park NX10 (Park Systems) atomic force microscope were used to obtain high resolution digital images of the mRNA-NPs. The samples for SEM were coated with Pt. The AFM samples (5 μl) were dissolved in 30 μl buffer (50 mM NiCl_2_ in TE buffer (pH 7.5; Integrated DNA Technologies)), and deposited onto freshly cleaved mica (Ted Pella). After incubation for 40 min, the mica surface was washed with Milli-Q water and dried. The samples were scanned in non-contact mode with NC-NCH tips (Park Systems).

### Serum digestion of mRNA-NPs

40 ng of the capped mRNA encoding Xef-1 (1800 bp) and mRNA-NPs (17.2 ng) were incubated with 2% or 10% FBS for 5 min or 1 h at 37 °C and inactivated by heating for 20 min at 70 °C. Gel electrophoresis was carried out on a 2% agarose gel at 95 V for 60 min to analyze the degradation of the control mRNA and mRNA-NPs. The size distribution of the serum digested free mRNA and mRNA-NPs was also analyzed by DLS.

### Dynamic light scattering and zeta potential analysis

The size and surface charge of the mRNA-NP, TransIT-X2 and TransIT-X2 covered mRNA-NP were measured using a Malvern Zetasizer Nano-ZS90 and analyzed with Zetasizer software (Malvern Instruments). Each sample was prepared immediately before use and diluted in nuclease-free water. All measurements were carried out at 25 °C, and three measurements with at least 10 sub-runs were performed for each sample.

### Labeling of mRNA

To label the mRNA-NPs, all the reaction components were mixed with different amounts of Cyanine 3-UTP (Enzo). The resulting solution after transcription of the mRNA-NPs was run in a 1.2% agarose gel at 100 V at room temperature in Tris-acetate-EDTA (TAE) buffer (40 mM Tris-acetate and 1 mM EDTA, pH 8.0, Biosesang) for 85 min. The gel was stained with GelRed (Biotium) in TAE buffer and examined immediately under ultraviolet light. The gel electrophoresis images were compared with each other to confirm the appropriate labeling of Cy3-UTP.

### Image cytometry

The TransIT-X2 covered mRNA-NP-transfected cells were assessed for GFP expression by image cytometry. Briefly, the cells were trypsinized and diluted with DPBS. Hoechst 33342 (ChemoMetec) at a final concentration of 10 μg ml^−1^ was added directly and incubated at 37 °C for 15 min. Propidium iodide (ChemoMetec) at a final concentration of 10 μg ml^−1^ was then added prior to image cytometry on a Nucleocounter (NC-3000, ChemoMetec) to assess the cell viability and GFP expression level. After staining, the cells were loaded into NC-Slide A2 (ChemoMetec). The samples were analyzed using NucleoView NC-3000 (ChemoMetec) software to determine the transfection ratio of the viable and nonviable cells, GFP mean intensity and estimated viability.

### Cell culture

PC-3 cells (kindly provided by the Korea Institute of Science and Technology) were grown in RPMI 1640 (Welgene) supplemented with 10% fetal bovine serum (Gibco), 100 units ml^−1^ of penicillin, 100 μg ml^−1^ of streptomycin, and 1% antibiotic-antimycotic (Gibco) at 37 °C in a humidified atmosphere supplemented with 5% CO_2_. The cells were passaged routinely to maintain exponential growth. Twenty-four hours before transfection (60–80% confluence), the cells were trypsinized, diluted with fresh medium (3 × 10^5^ cells ml^−1^) and transferred to 24-well plates (500 μl per well).

### Covering mRNA-NP with TransIT-X2 for transfection

To cover the mRNA-NPs with the TransIT-X2 Delivery System (Mirus), the mRNA-NPs were diluted with OPTI-MEMI (Gibco). TransIT-X2 was mixed gently into the reaction solution, and the mixtures were incubated at room temperature for 15 min according to the manufacturer’s instructions.

### Transfection of the mRNA-NPs

Each concentration of the TransIT-X2 covered mRNA-NPs was diluted for each well of the cells, and the cells were incubated for 3–48 hours at 37 °C in a humidified atmosphere supplemented with 5% CO_2_.

### Imaging GFP expressing PC-3 cells

To image GFP expressing PC-3 cells, the cells were grown on an 8-well cell culture chamber slides (SPL Life Science). Eclipse Ti (Nikon) inverted fluorescent microscopy was used to image the transfected cells. All the cells were fixed with 4% paraformaldehyde (MBiotech), and stained with DAPI at a final concentration of 5 μg ml^−1^ to locate the cell nucleus. Prior to each step, the cells were washed twice with Dulbecco’s phosphate buffered saline (DPBS, Gibco).

## Additional Information

**How to cite this article**: Kim, H. *et al*. Self-assembled Messenger RNA Nanoparticles (mRNA-NPs) for Efficient Gene Expression. *Sci. Rep*. **5**, 12737; doi: 10.1038/srep12737 (2015).

## Supplementary Material

Supplementary Information

## Figures and Tables

**Figure 1:**
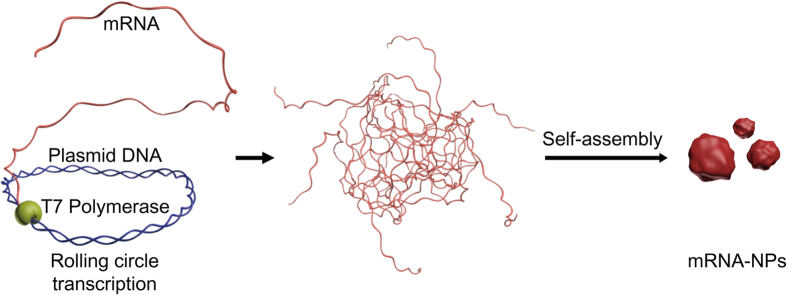
Schematic diagram outlining the synthesis of mRNA-NPs. T7 RNA polymerases enzymatically transcribe mRNA strands via rolling circle transcription reaction with the plasmid DNA as the template DNA. The resulting multiple copies of the mRNA strands were entangled and self-assembled into nanoscopic particles.
