# Supplementary material for: Self-assembled Messenger RNA Nanoparticles (mRNA-NPs) for Efficient Gene Expression
Source: Sci Rep. 2015 Aug 3;5:12737. doi: 10.1038/srep12737 (PMC4522648; doi:10.1038/srep12737)
Supplement: Supplementary Information [file srep12737-s1.pdf]

Supplementary Information

Self-assembled Messenger RNA Nanoparticles (mRNA-NPs)

for Efficient Gene Expression

Hyejin Kim[1], Yongkuk Park[1] and Jong Bum Lee[1,*]

[1]Department of Chemical Engineering, University of Seoul, Seoul 130-743, Republic of Korea

*Fax: +82-2-6490-2364; Tel: +82-2-6490-2372; E-mail: jblee@uos.ac.kr

Correspondence and requests for materials should be addressed to J.B.L.

Supplementary Methods

Determination of optimal concentrations of RCT reaction components

RCT reaction was carried out with different concentrations of rNTPs (0.5 mM, 2.5 mM, 5.0 mM and 10.0 mM), T7 RNA polymerase (5.00 U μl^{-1}, 2.50 U μl^{-1}, 1.67 U μl^{-1}, 1.25 U μl^{-1}) or plasmid DNA (1.0 nM, 0.5 nM and 0.1 nM) were analyzed with gel electrophoresis. RCT products were run in 1.2% agarose gel at 100 V at room temperature in Tris-acetate-EDTA (TAE) buffer (40 mM Tris-acetate and 1 mM EDTA, pH 8.0, Biosesang) for 85 min. The gel was stained with GelRed (Biotium) in TAE buffer and examined immediately under ultraviolet light.

Molecular weight analysis

Molecular weight analysis was based on the fluorescence-based determination of mass concentration and image cytometry analysis. First, the mass concentration of RNA generated is determined by fluorescence-based method. Briefly, four different dilutions of single-stranded RNA (New England Biolabs) are stained with SYBR Green II for the standard curve. Fluorescent intensities of each concentration of ssRNA were then measured with microplate reader (ex. 485/20, em. 528/20). Then, three different dilutions of mRNA-NP-1 were sonicated for 5 min, and stained with SYBR Green II. The RNA concentrations of the samples were determined from the standard curve generated. For the determination of molar concentrations of mRNA-NPs, mRNA-NPs were stained with DAPI. Stained particles were analyzed by image cytometry (Nucleocounter NC-3000) according to the manufacturer's instruction. The molecular weight of mRNA-NP-1 was then calculated by simply dividing mass concentration by molar concentration. Number of bases of RNA generated in a single mRNA-NP was obtained by dividing the calculated molecular weight by the molecular weight of 2855 base

RNA strand (from 2855 base plasmid DNA template) is 918374 g mol^{-1}, then multiplying by 2855.

Synthesizing and transfection of mRNA-NP-1 with a scrambled sequence

mRNA-NP-1 encoding firefly luciferase was tested as a control with a scrambled sequence. Rolling circle transcription was carried out with pCMV-Luc (5566 bp) as template plasmid DNA with the same condition for fabricating mRNA-NP-1 encoding GFP (mRNA-NP-1-GFP). Resulting luciferase-encoding mRNA nanoparticles (mRNA-NP-1-Luc) were confirmed with DLS (165.3 ± 30.78 nm), then PC-3 cells were treated with 0.6 fM of mRNA-NP-1-Luc.

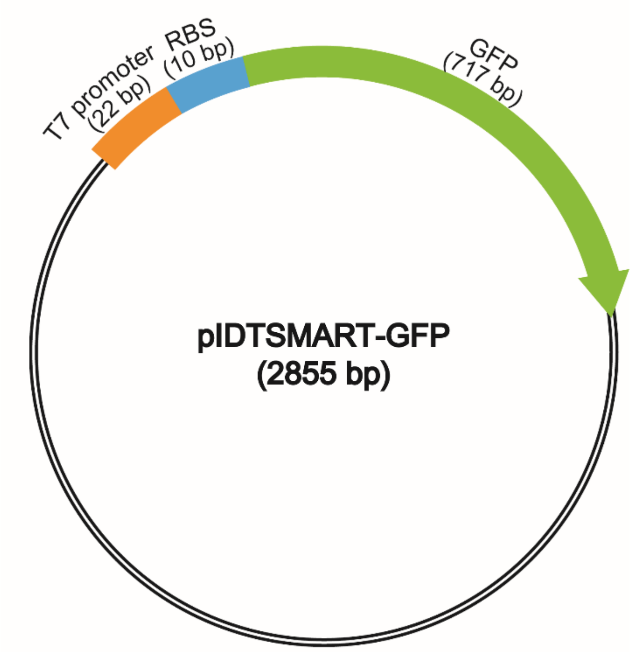

Figure S1. Plasmid map of pIDT-SMART-GFP (2855 bp) encoding GFP (green region), ribosomal binding site (blue region) and T7 promoter region (orange region) for RCT reaction.

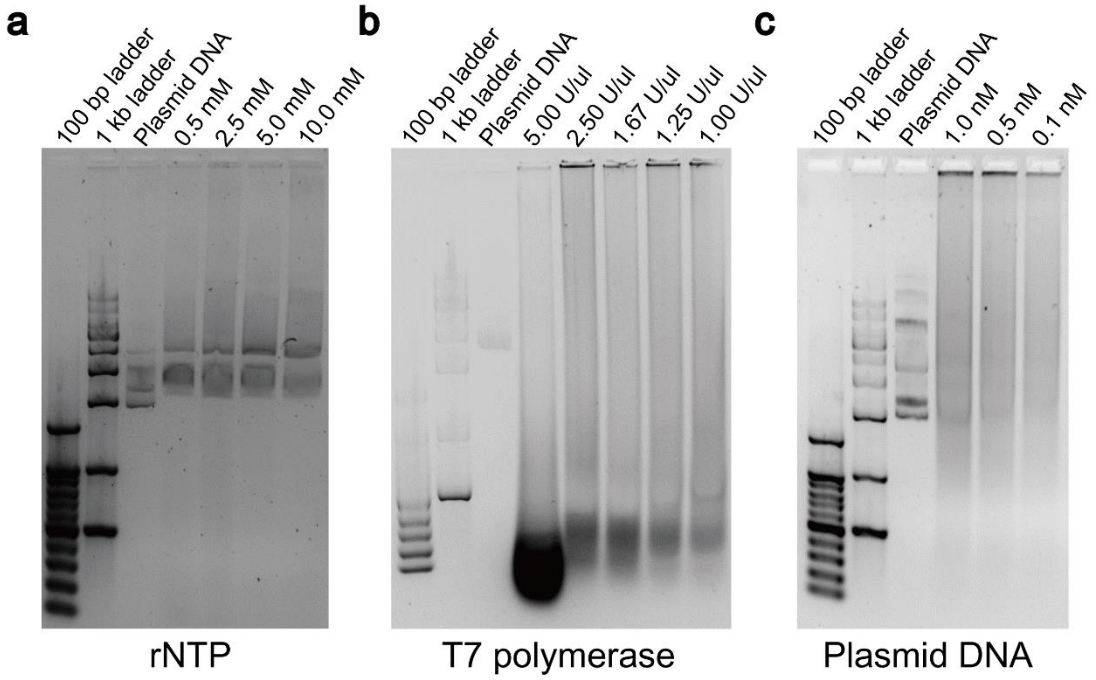

Figure S2. Determination of optimal concentrations of RCT reaction components. Gel electrophoresis analysis of the products of RCT reaction with different concentrations of rNTPs (a), T7 polymerase (b) and plasmid DNA (c), respectively. The resulting mRNA-NPs were trapped within each well of the gel. And lanes 1-3 indicate 100 bp DNA ladder, 1 kb DNA ladder and plasmid DNA, respectively. a) Lanes 4-7 indicate products of RCT reaction carried out with 0.5 mM, 2.5 mM, 5.0 mM and 10.0 mM, respectively. Concentrations of rNTPs had no significant effect on RCT reaction with concentrations ranging from 0.5 to 10.0 mM. b) Lanes 4-8 indicate products of RCT reaction carried out with 5.00 U μl^{-1}, 2.50 U μl^{-1}, 1.67 U μl^{-1}, 1.25 U μl^{-1} and 1.00 U μl^{-1} of T7 RNA polymerase, respectively. RCT reaction with 5 U μl^{-1} of T7 RNA polymerase resulted in low efficiency of mRNA-NP synthesis. c) Lanes 4-6 indicate products of RCT reaction carried out with 1.0 nM, 0.5 nM and 0.1 nM of plasmid DNA, respectively. Concentrations of plasmid DNA lower than 0.1 nM resulted in low efficiency of RNA synthesis. Gel electrophoresis was carried out on a 1% agarose gel for the analysis (a-c).

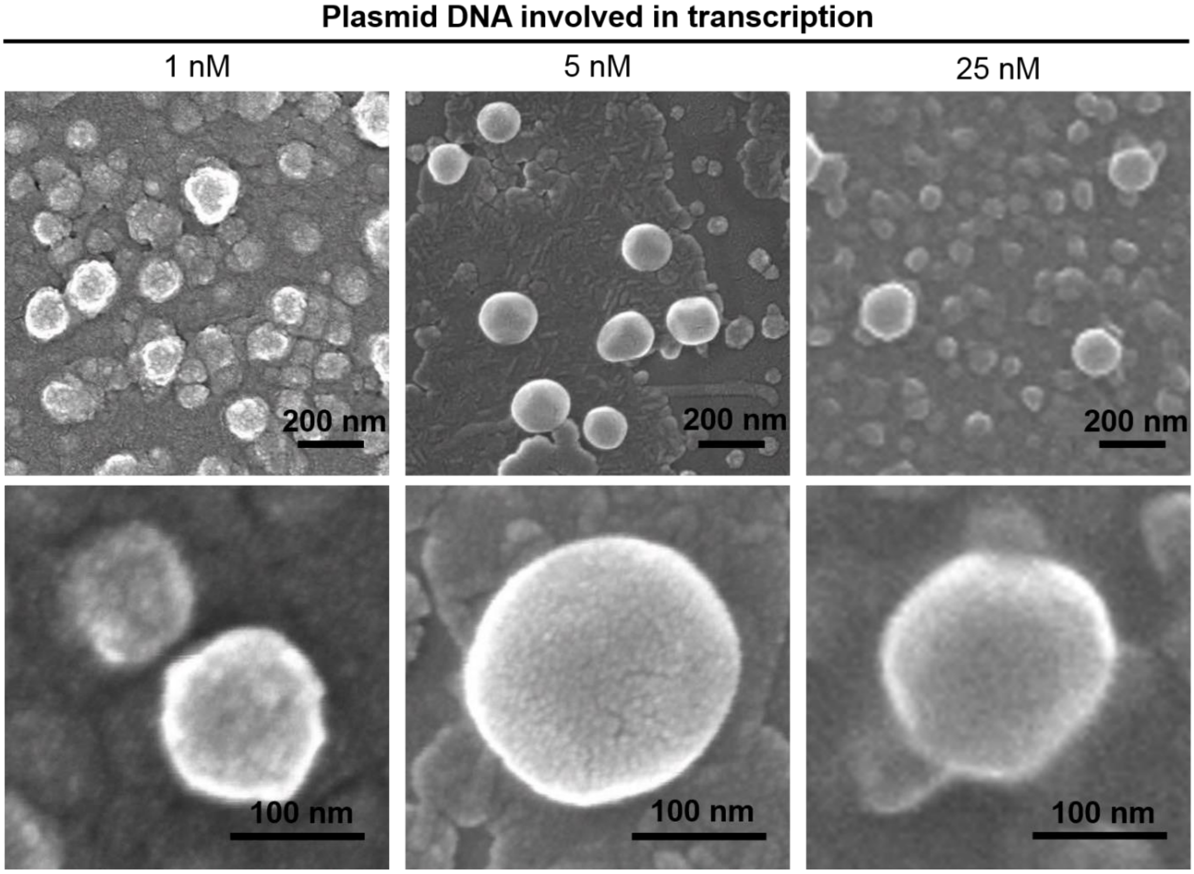

Figure S3. SEM images of mRNA-NPs transcribed from 1 nM (left), 5 nM (middle) and 25 nM (right) of plasmid DNA, respectively.

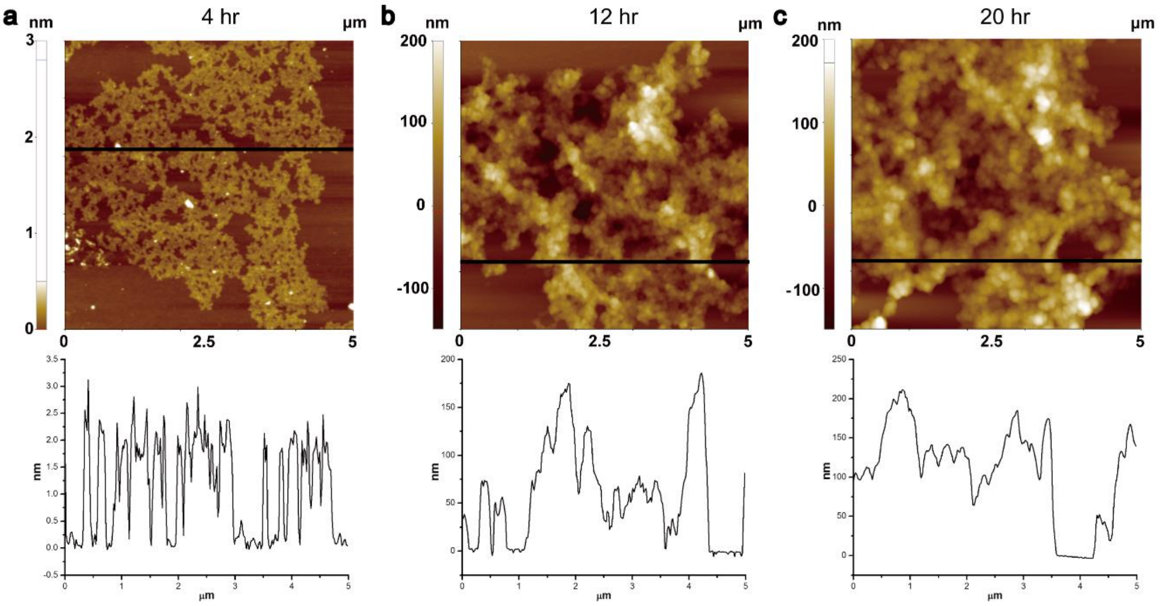

Figure S4. Time-course of mRNA-NP formation and cross-sectional analysis. After 4 h (a), 12 h (b) and 20 h (c) of RCT, respectively. The result reveals that the height of entangled mRNA strands is only about 2 nm (a), indicating RCT products have two dimensional structure at the early stage of the reaction. After further reaction time, RCT products started to form three-dimensional structures with the formation of interconnected bead-like small particles having diameters of about 30 nm (b). After 20 h of RCT reaction, cross-sectional analysis reveals that the resulting globular structures are about 200 nm in diameter (c).

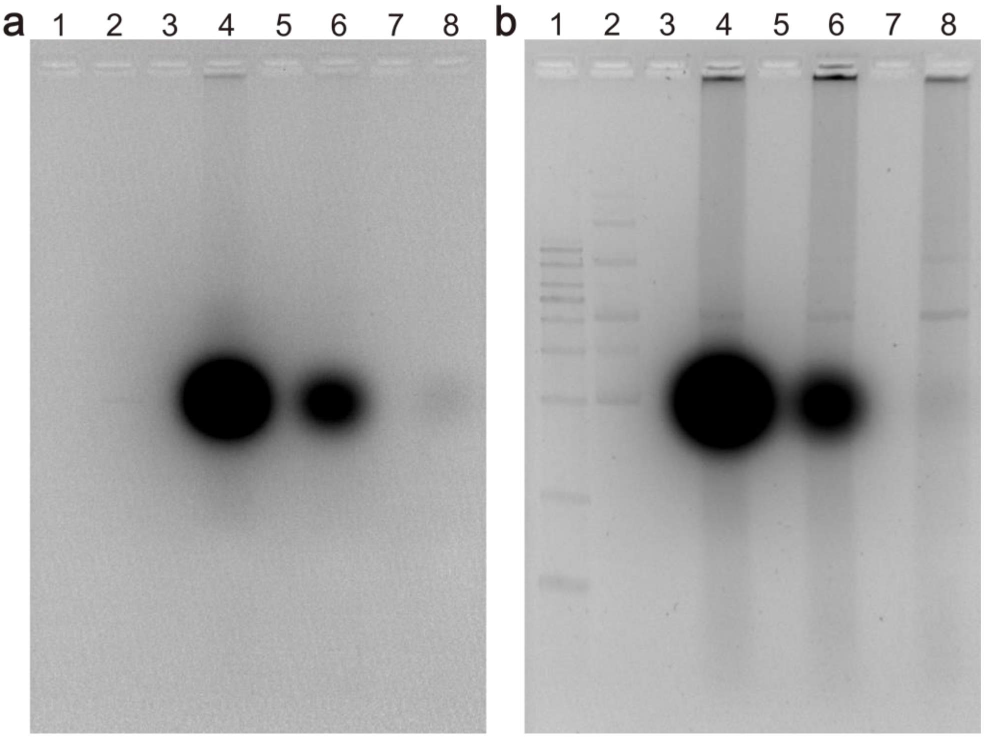

Figure S5. Gel electrophoresis results after 20 hours of RCT with different concentrations of Cy3-UTP. Gel image of RCT products revealing green fluorescence of Cy3 under ultraviolet light before staining (a) and after staining with GelRed (c). mRNA-NPs were entrapped within each well of the gel and were visible without staining (a; lane 4, 6 and 8). Lanes 1 and 2 indicate 1 kb DNA ladder and plasmid DNA, respectively. Lanes 4, 6 and 8 correspond to the RCT reaction with 100 μM, 20 μM and 5 μM of Cy3-UTP, respectively. Gel electrophoresis was carried out on a 1% agarose gel for the analysis (a and b).

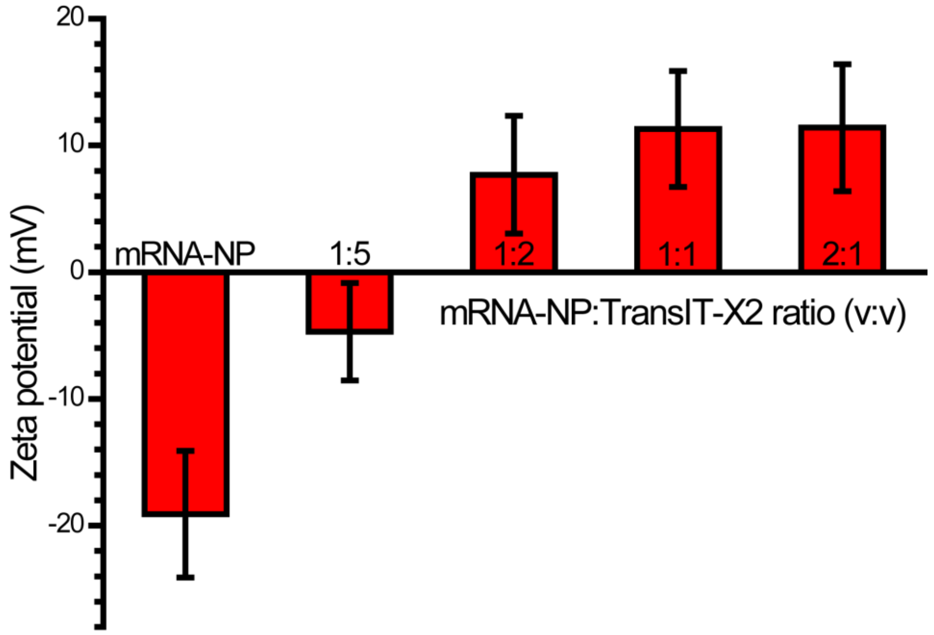

Figure S6. Zeta potential analysis of mRNA-NPs and TransIT-X2 coated mRNA-NPs at different volume ratio. mRNA-NP alone was negatively charged (-19.1 mV), and zeta potential of complexes formed with mRNA-NP:TransIT-X2 volumetric ratio of 1:5, 1:2, 1:1 and 2:1 were tested.

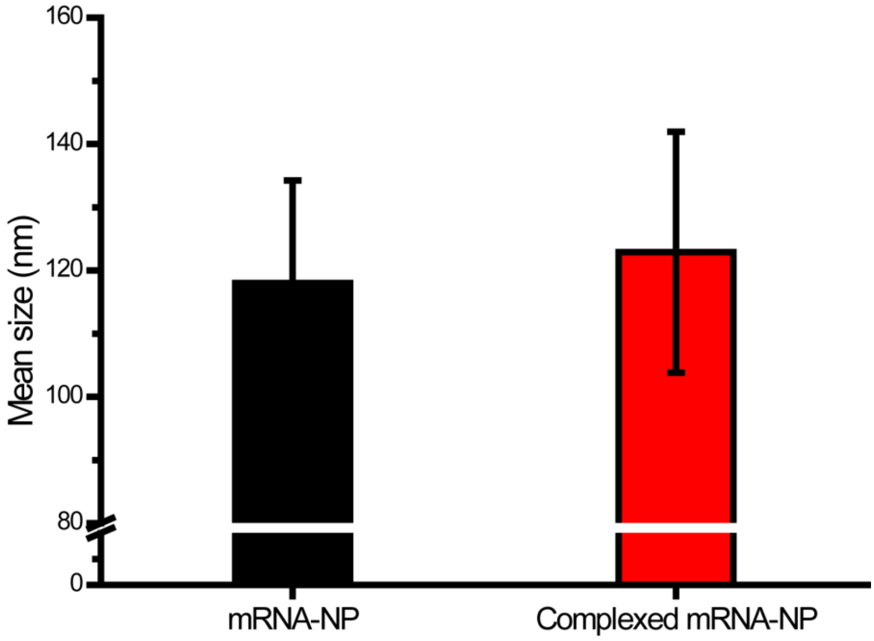

Figure S7. Size distributions of mRNA-NP-1 (black) and mRNA-NPs coated with TransIT-X2 (red) with volumetric ratio of 1:1, respectively.

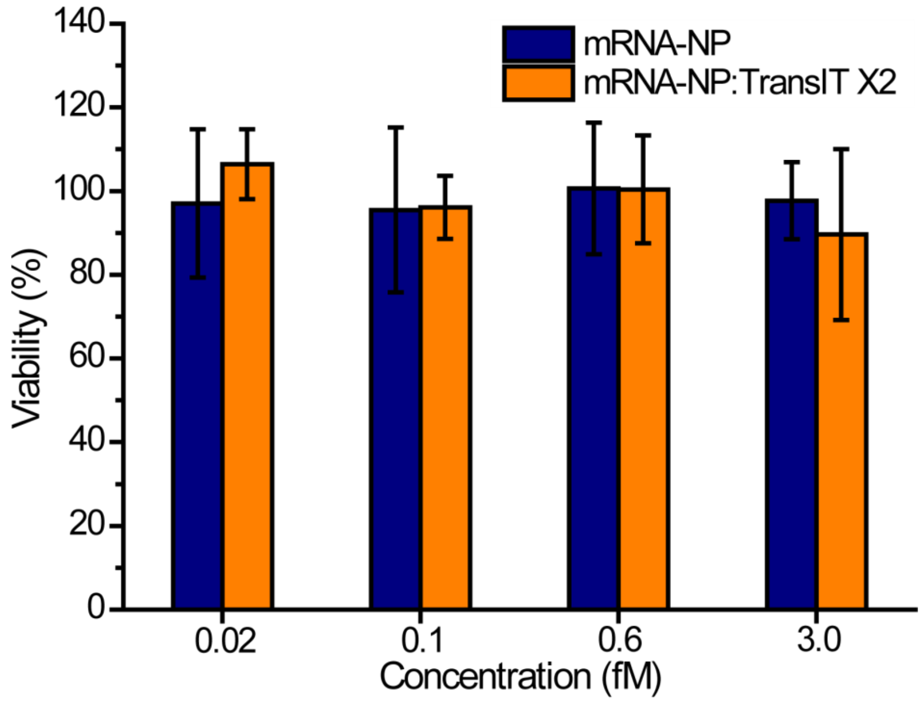

Figure S8. Cell viabilities of the PC-3 cells at 24 h after treated with the mRNA-NP-1 (navy) or coated mRNA-NP-1 with transfection agent (orange). Each well of PC-3 cells was treated with 0.02 fM, 0.1 fM, 0.6 fM and 3.0 fM of the mRNA-NP-1 or coated mRNA-NP-1, respectively. mRNA-NP-1 alone at all concentrations and the coated mRNA-NP-1 at concentrations of 0.02 fM, 0.1 fM and 0.6 fM did not significantly affect the viability of treated cells, indicating that the mRNA-NPs have low cytotoxicity. This result also suggests that non-toxic amounts of transfection agent were required to achieve efficient cellular uptake of mRNA-NPs. In the manuscript, 0.6 fM of mRNA-NP-1 was used to demonstrate subcellular expression of GFP.

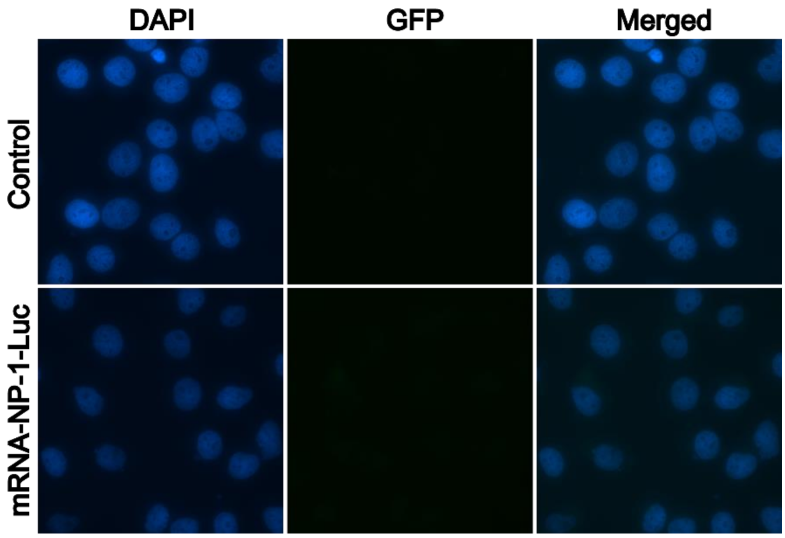

Figure S9. Fluorescent images of PC-3 cells 24 h after transfection of mRNA-NPs with a scrambled sequence. Firefly luciferase encoding plasmid DNA was used as a template DNA, and the cells after treatment with mRNA-NP-1-Luc showed no green fluorescence intensity.

Table S1. Size distribution of mRNA-NPs after serum digestion, analyzed with dynamic light scattering. mRNA-NPs were treated with 2% or 10% nuclease-containing serum for 5 min or 1 h or left untreated as a control. Mean size of the particle decreased with the treatment of higher amount of serum or longer incubation time, indicating mRNA-NPs were degraded by nucleases contained within FBS. However, particle formation of mRNA-NPs was still retained even after 10% serum digestion for 1 h.

FBS (%)	Incubation time (min)	Mean size (nm)
Control	-	117.1 ± 22.18
2	5	112.1 ± 64.51
	60	73.59 ± 38.72
10	5	74.66 ± 33.88
	60	69.48 ± 35.34

Table S2. Molecular weights of mRNA-NPs and the length of RNA generated within one mRNA-NP when different molar concentrations of plasmid DNA involved in RCT reaction.

**Plasmid DNA involved in RCT reaction (nM)**	**Molecular weight of mRNA-NP (g mol^{-1})**	**RNA in a single mRNA-NP (megabases)**
1.0	2.30×10^{10}	71.5
5.0	3.77×10^{10}	117.2
